# Randomized placebo-controlled double-blind phase II study of zaltoprofen for patients with diffuse-type and unresectable localized tenosynovial giant cell tumors: The REALIZE study

**DOI:** 10.3389/fonc.2022.900010

**Published:** 2022-09-21

**Authors:** Akihiko Takeuchi, Makoto Endo, Akira Kawai, Yoshihiro Nishida, Ryu Terauchi, Akihiko Matsumine, Hisaki Aiba, Tomoki Nakamura, Susumu Tandai, Toshifumi Ozaki, Manabu Hoshi, Daiki Kayano, Miho Okuda, Norio Yamamoto, Katsuhiro Hayashi, Shinji Miwa, Kentaro Igarashi, Kenichi Yoshimura, Akihiro Nomura, Toshinori Murayama, Hiroyuki Tsuchiya

**Affiliations:** ^1^Department of Orthopaedic Surgery, Kanazawa University Graduate School of Medical Sciences, Kanazawa, Japan; ^2^Department of Orthopaedic Surgery, Graduate School of Medical Sciences, Kyushu University, Fukuoka, Japan; ^3^Department of Musculoskeletal Oncology, National Cancer Center Hospital, Tokyo, Japan; ^4^Department of Orthopaedic Surgery, Nagoya University School of Medicine, Nagoya, Japan; ^5^Department of Orthopaedic Surgery, Kyoto Prefectural University of Medicine, Kyoto, Japan; ^6^Department of Orthopaedics and Rehabilitation Medicine, Unit of Surgery, Division of Medicine, Faculty of Medical Sciences, University of Fukui, Fukui, Japan; ^7^Department of Orthopaedic Surgery, Nagoya City University Graduate School of Medical Sciences, Nagoya, Japan; ^8^Department of Orthopedic Surgery, Mie University School of Medicine, Mie, Japan; ^9^Department of Orthopaedic Surgery, Asahikawa Medical University, Hokkaido, Japan; ^10^Department of Orthopedic Surgery, Okayama University Graduate School of Medicine, Dentistry, and Pharmaceutical Sciences, Okayama, Japan; ^11^Department of Orthopedic Surgery, Osaka City University Graduate School of Medicine, Osaka, Japan; ^12^Department of Nuclear Medicine, Kanazawa University Hospital, Kanazawa, Japan; ^13^Department of Radiology, Kanazawa University Graduate School of Medical Sciences, Kanazawa, Japan; ^14^Future Medical Center, Hiroshima University Hospital, Hiroshima, Japan; ^15^Innovative Clinical Research Center (iCREK), Kanazawa University Hospital, Kanazawa, Japan

**Keywords:** tenosynovial giant cell tumor (TGCT), zaltoprofen, nonsteroid anti-flammatory drugs, randomizad controlled trial, clinical trial

## Abstract

**Background:**

A tenosynovial giant cell tumor (TGCT) is a locally aggressive benign neoplasm arising from intra- or extra-articular tissue, categorized as localized (L-TGCT, solitary lesion) and diffuse (D-TGCT, multiple lesions) TGCT. Surgical excision is the mainstay of the treatment, and a high local recurrence rate of approximately 50% has been reported. We focused on zaltoprofen, a nonsteroidal anti-inflammatory drug that can activate peroxisome proliferator-activated receptor gamma (PPARγ) and inhibit the proliferation of TGCT stromal cells. Therefore, we conducted a randomized trial to evaluate the safety and effectiveness of zaltoprofen in patients with D-TGCTs or unresectable L-TGCTs.

**Methods:**

This randomized, placebo-controlled, double-blind, multicenter trial evaluated the safety and efficacy of zaltoprofen. In the treatment group, zaltoprofen (480 mg/day) was administered for 48 weeks; the placebo group received similar dosages without zaltoprofen. The primary outcome was progression-free rate (PFR) 48 weeks after treatment administration. Disease progression was defined as the following conditions requiring surgical intervention: 1) repetitive joint swelling due to hemorrhage, 2) joint range of motion limitation, 3) invasion of the adjacent cartilage or bone, 4) severe joint space narrowing, and 5) increased tumor size (target lesion).

**Results:**

Forty-one patients were allocated to the zaltoprofen (n=21) or placebo (n=20) groups. The PFR was not significant between the zaltoprofen group and the placebo group at 48 weeks (84.0% and 90.0%, respectively; p=0.619). The mean Japanese Orthopedic Association knee score significantly improved from baseline to week 48 in the zaltoprofen group (85.38 versus 93.75, p=0.027). There was a significant difference between the values at 48 weeks of placebo and zaltoprofen group (p=0.014). One severe adverse event (grade 3 hypertension) was observed in the zaltoprofen group.

**Discussion:**

This is the first study to evaluate the efficacy and safety of zaltoprofen in patients with TGCT. No significant differences in PFR were observed between the groups at 48 weeks. Physical function significantly improved after zaltoprofen treatment. The safety profile of zaltoprofen was acceptable. This less invasive and safer treatment with zaltoprofen, compared to surgical removal, could be justified as a novel approach to treating TGCT. Further analysis of long-term administration of zaltoprofen should be considered in future studies.

**Clinical Trial Registration:**

University Hospital Medical Information Network Clinical Trials Registry, identifier (UMIN000025901).

## 1 Introduction

A tenosynovial giant cell tumor (TGCT) is a locally aggressive benign soft tissue tumor arising from various joints. A localized TGCT (L-TGCT), previously called a giant cell tumor of the tendon sheath, usually arises from small joints, including the hand and foot. It is slightly more predominant in women than in men (1) and its annual incidence has been reported as approximately 1 in 50,000 (2). A diffuse TGCT (D-TGCT) is synonymous with pigmented villonodular synovitis that develops in large joints, including the knee, hip, ankle, elbow, and shoulder. An annual incidence of approximately 2 cases per 1 million has been reported in individuals aged <40 years with a slight female predominance (3).

Surgical excision (open or arthroscopic synovectomy) is the standard treatment for TGCTs, but the local recurrence rate has been reported to be 16–47% (4, 5). Additionally, the pathogenesis of TGCT has been attributed to the overexpression of colony-stimulating factor-1 (CSF-1) due to fusion of the *CSF1* gene to the collagen type VI α3 promoter in the t (1, 2) translocation (6). Therefore, systemic therapies targeting the CSF-1/colony-stimulating factor-1 receptor (CSF-1R) axis have been developed (7). Pexidartinib is the first approved systemic therapy for patients with TGCT in the United States (8). It was reported that the favorable response rate, however it also needs the risk assessment including the hepatotoxicity (9). In contrast, other therapeutic agents have also been proposed that have shown promise in the treatment of TGCTs. Specifically, we previously analyzed the anti-tumor effect of zaltoprofen, a nonsteroidal anti-inflammatory drug (NSAID), *via* peroxisome proliferator-activated receptor gamma (PPARγ) in some musculoskeletal tumors, including TGCT (10), giant cell tumor of bone (11) and chondrosarcoma (12). Zaltoprofen is traditionally used and indicated for joint disorders, rheumatoid arthritis (13), and neuropathic pain (14). PPARγ is a key transcriptional factor that stimulates adipocyte differentiation (15). It also exhibits antitumor activity by inhibiting tumor proliferation and invasion and through the induction of differentiation and apoptosis. PPARγ ligands, including synthetic ligands, such as thiazolidinedione (16) and 15-deoxy-delta-12,14-prostaglandin J2 (17) have been investigated. Certain NSAIDs, including indomethacin, act as direct ligands for PPARγ (18). Zaltoprofen has been reported to induce apoptosis in rheumatoid synovial cells *via* PPARγ activation (19). Clinical trials of targeted therapies for PPARγ have been reported in some types of cancer, including liposarcoma (20), breast cancer (21), prostate cancer (22), and colon cancer (23).

The long-term safety of zaltoprofen in patients with rheumatoid arthritis has been previously reported (13). We also performed a pilot study of zaltoprofen treatment in 10 patients (6 knees and 4 ankles) for diffuse-type TGCTs. Eight of these patients (80%) maintained stable disease (SD) at 48 weeks, while one patient (10%) showed progressive disease (PD) at 72 weeks. These results suggest that zaltoprofen could be expected to maintain a stable disease and alternative treatment option for TGCT (24). However, there was no clinical trial with this drug for TGCT. Therefore, we conducted a randomized clinical trial to evaluate the efficacy and safety of zaltoprofen in patients with D-TGCTs or unresectable L-TGCTs (the REALIZE study) (25).

## 2 Materials and methods

### 2.1 Ethics statements

This trial was designed by the investigators to evaluate the safety and efficacy of zaltoprofen in patients with D-TGCTs or L-TGCTs and was approved by the Pharmaceuticals and Medical Devices Agency (PMDA). The Center for Clinical Trials, Japan Medical Association (JMACCT), and Japan Agency for Medical Research and Development (AMED) funded this trial (JMA-IIA00284 and 21lk0201120h0002). The trial network consisted of a lead site at the Innovative Clinical Research Center, Kanazawa University (iCREK) (Kanazawa, Japan), and 10 additional sites (Asahikawa Medical University Hospital; National Cancer Center Hospital; Fukui University Hospital; Nagoya City University Hospital; Nagoya University Hospital; Mie University Hospital; University Hospital, Kyoto Prefectural University of Medicine; Osaka City University Hospital; Okayama University Hospital; and Kyusyu University Hospital) in Japan.

The study was conducted in compliance with the Declaration of Helsinki, International Conference on Harmonisation (ICH)-Good Clinical Practice, and all other laws and guidelines applicable to Japan. The protocol was approved by the institutional review boards (IRBs) of Kanazawa University Hospital and each participating hospital. This study was registered with the University Hospital Medical Information Network (UMIN) Clinical Trials Registry (UMIN000025901). Written informed consent was obtained from all trial participants. The consent forms were approved by the IRB of each center.

### 2.2 Study design and definitions

The protocol of this randomized, placebo-controlled, double-blind, multicenter study, which was conducted from July 2017 to June 2019, as previously reported (25); and zaltoprofen (480 mg/day) was administered for 48 weeks in the treatment group.

Disease progression was defined as the following conditions requiring surgical intervention: 1) the joint circumference was increased by ≥2 cm with respect to the baseline (knee, 1 cm above the patella; ankle joint, determined using the figure-eight method). If there was fluid accumulation, the presence or absence of a hematoma was examined. An increase in joint circumference due to edema was not considered an exacerbation. 2) The range of motion of the joint (i.e., active motion) was reduced by ≥20% with respect to the baseline (calculated by averaging three measurements with a goniometer). Condition 1 or 2 must be detected in two consecutive evaluation periods conducted every 4 weeks. 3) An invasion of ≥5 mm of bone/cartilage erosion or a new lesion of bone or cartilage erosion ≥5 mm, compared with that at baseline, was detected by computed tomography or magnetic resonance imaging. 4) X-ray in the standing position shows disappearance of the joint space. 5) An increase in the target lesion by ≥20% was determined by the local investigator and a central committee radiologist. Condition 3, 4, or 5 must be detected once in any evaluation period conducted every 12 weeks. 6) Any surgical intervention for a disease that is performed for a specific reason.

### 2.3 Study participants

The inclusion criterion was patients aged 20–70 years who were diagnosed with D-TGCT or unresectable L-TGCT occurring in the knee joint or ankle based on radiological and pathological findings, measurable based on Response Evaluation Criteria in Solid Tumors (RECIST) (version 1.1) (26), and maintaining joint space on standing X-ray imaging. Patients were excluded if they had severe heart disease, renal disease, respiratory disease, blood disease, diabetes, coagulopathy, hepatic injury, gastric ulcers, or severe joint disorders due to tumor progression. Details of the inclusion and exclusion criteria are shown in **Supplementary Tables 1****,** **2**.

### 2.4 Randomization

The participants were randomly assigned using a computer-generated random sequence with stratification for the size of tumor lesion and tumor location (i.e., knee or foot joints) at a 1:1 ratio to receive zaltoprofen or placebo.

### 2.5 Intervention and placebo

In the treatment (intervention) group (group Z), patients received two oral tablets of zaltoprofen (80 mg per tablet) three times daily. In the placebo group (group P), patients received two oral placebo tablets three times daily.

### 2.6 Outcomes

The primary outcome of this study was the progression-free rate (PFR) based on the original criteria 48 weeks after treatment administration. The secondary endpoints were as follows: 1) PFR based on the RECIST criteria (24 weeks and 48 weeks), as assessed by the local investigator and central committee radiologist; 2) maximum standardized uptake value (SUVmax) change by ^18^F-fluorodeoxyglucose-positron emission tomography (PET); 3) affected limb function (baseline, 24 weeks, and 48 weeks) according to the Japanese Orthopedic Association (JOA) score and Musculoskeletal Tumor Society (MSTS) score; 4) percentage of cases in which clinical benefit (pain, range of joint motion, and joint function) was obtained at 24 and 48 weeks, as judged by investigators or clinical trial physicians); and 5) adverse events graded using Common Terminology Criteria for Adverse Events version 4.0.

The JOA score for knee osteoarthritis was established in Japan to evaluate knee function, and it consists of four factors: pain on walking (30 points), pain on ascending or descending stairs (25 points), mobility (35 points), and joint effusion (10 points). Higher scores indicate better function. Meanwhile, the JOA Foot Rating Scale was established in Japan to evaluate foot and ankle functions. It comprises three major points: pain (40 points), function (50 points), and alignment (10 points). Function consists of activity limitation, maximum continuous walking distance, walking surfaces, gait abnormality, sagittal motion, hindfoot motion, and ankle-hindfoot stability. The total score ranges from 0 to 100, with higher scores indicating better function (27). The MSTS Rating Scale was developed in 1983 and modified by the MSTS in 1993 (28). It comprises six factors: pain, function, emotional acceptance, use of any external support, walking ability, and gait alteration. Each factor is rated on a scale of 0–5. The total score ranges from 0% to 100%, with higher scores indicating better function.

### 2.7 Safety assessment

The investigator at each institution reported any adverse events according to the ICH guideline-E6 and E2A (https://www/.ich.org/home.html).

### 2.8 Sample size

Based on our estimation of the PFRs as 80% and 30% in groups Z and P, respectively, we used the log-rank test to determine that 20 participants per group would be required for 95% confidence interval and power of 90% in the sample size calculation.

### 2.9 Statistical analysis

The primary endpoint was analyzed based on the full analysis set (excluding participants who did not meet the inclusion criteria or did not take the study drugs) and was compared between groups Z and P at 48 weeks using the log-rank test. The secondary endpoints were compared between the groups at baseline and at 24- and 48-weeks using t-tests, Mann-Whitney U tests, Fisher’s exact tests or Kruskal-Wallis tests. Statistical significance was set at p < 0.05. Statistical analyses were performed using SPSS version 25.0 (IBM Corp., Armonk, NY, USA).

## 3 Results

### 3.1 Demographics

**Figure 1:**
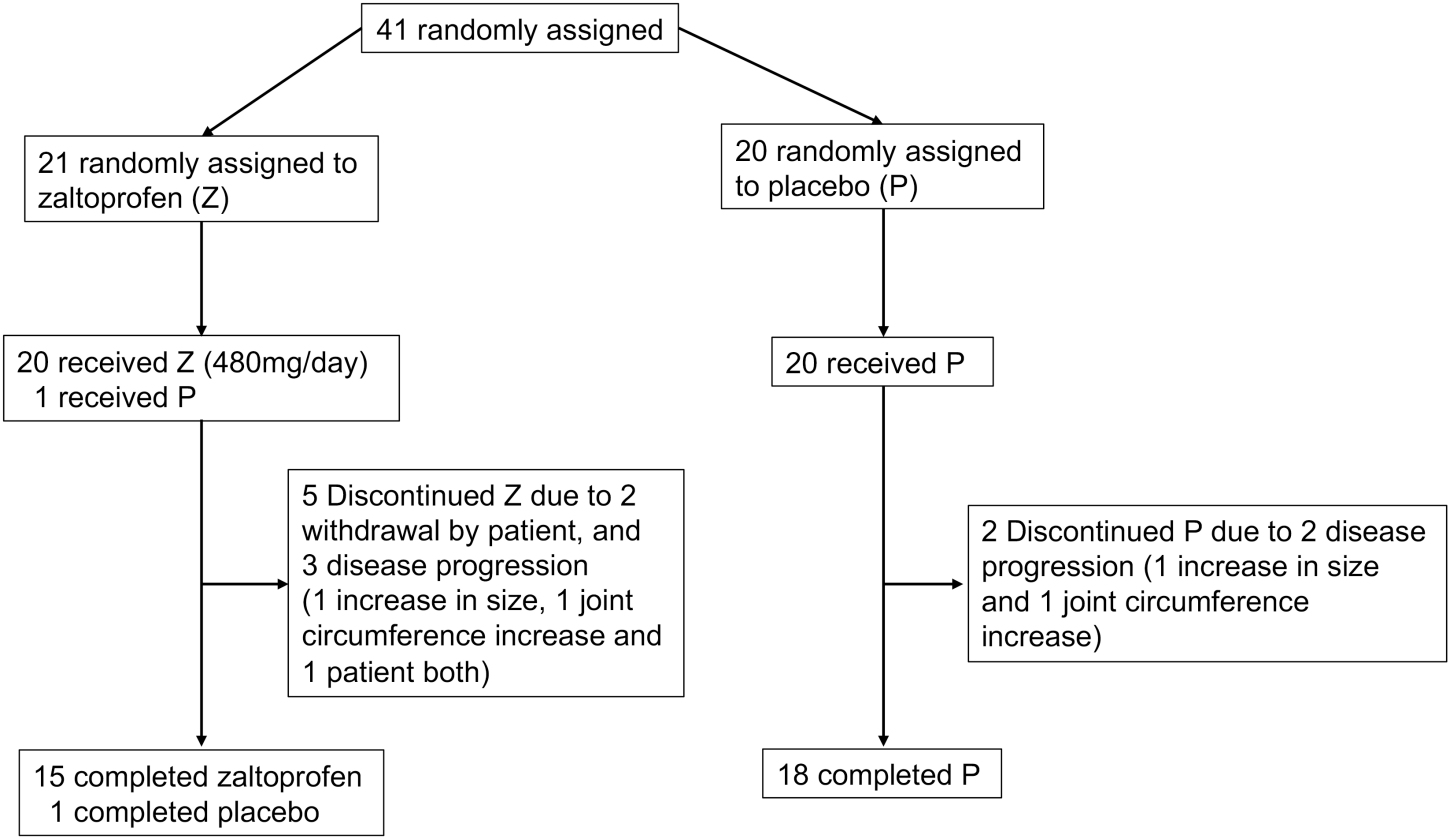
Flowchart of the selection process.

Forty-one patients were enrolled in this study. The patients’ mean age was 45.8 (range, 21–69 years), and there were 17 men and 24 women. The tumor sites were the knee in 26 patients and the ankle in 15 patients. Twenty-five patients received a previous surgery. None of the patients had received systemic therapy or radiotherapy. Few cases of concomitant spondylarthritis and TGCT have been reported (29), with no patient having concurrent rheumatoid arthritis. Twenty-one patients were randomly allocated to groups Z and P. Patient characteristics are shown in **Table 1**. One patient was allocated to group Z but received a placebo inadvertently. This patient was reviewed for efficacy in group Z and safety in group P. Two patients in group Z discontinued treatment because of their own wishes at 24 weeks (**Figure 1**).

**Table 1:** Patient characteristics.

		Zaltoprofen (n=21)	Placebo (n=20)
Sex	men	7	10
	women	14	10
Age (years)		45.0 (range, 21-66)	46.6 (range, 24-69)
Previous surgery	yes	13	12
	no	8	8
Location	knee	13	13
	Ankle	8	7
Mean size (mm)		102.3 (range, 21-243)	97.6 (range, 11-268)
(assessed by local investigator)			
Mean size (mm)		134.5 (range, 10-267)	131.5 (range, 30-282)
(assessed by central committee radiologist)			
Bone invasion	yes	13	11
	no	8	9
PET (SUVmax)		8.9 (range, 2.4-18.5)	10.5 (range, 2.4-25.1)
JOA score	knee	85.4 (range, 60-100)	83.4 (range, 70-95)
	ankle	83.6 (range, 67-100)	83.0 (range, 65-100)
MSTS score (%)		83.8 (range, 56.7-100)	82.2 (range, 50-100)

PET, positron emission tomography; SUV, Standardized Uptake Value; JOA, The Japanese Orthopedic Association; MSTS, Musculoskeletal Tumor Society.

### 3.2 Efficacy

#### 3.2.1 Primary endpoints

**Figure 2:**
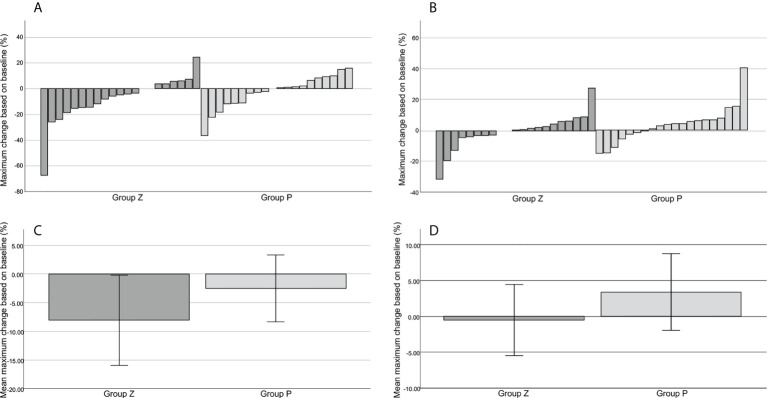
The best tumor shrinkage. Change from the baseline in the target tumor size assessed by the local investigator **(A)**. Change from the baseline in the target tumor size assessed by the central committee radiologist **(B)**. The mean reduction rate of the target lesion assessed by the local investigator **(C)**. The mean reduction rate of the target lesion assessed by the central committee radiologist **(D)**. The mean reduction rates of the target lesion were -8.03% ± 3.93 (mean ± SE) in group Z and -2.53% ± 2.92 (mean ± SE) in group P (p=0.271), as judged by the local investigator **(C)**, and -0.53% ± 2.49 (mean ± SE) in group Z and 3.38% ± 2.68 (mean ± SE) in group P (p=0.292), as judged by the central committee radiologist **(D)**. Group Z, zaltoprofen group; group P, placebo group; SE, standard error.

**Figure 3:**
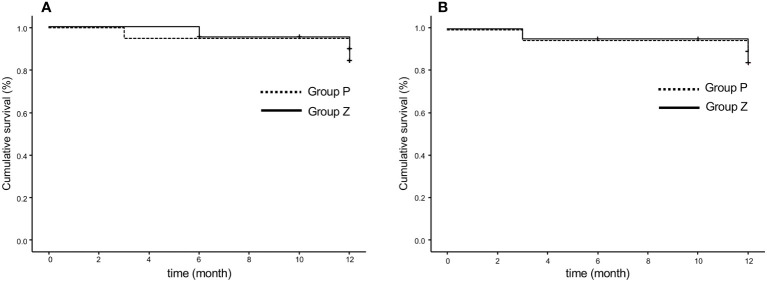
Progression-free rate based on the original criteria assessed by the local investigator **(A)** and central committee radiologist **(B)**. The progression-free survival rates were 84.0% in group Z and 90.0% in group P at 48 weeks (p=0.619) **(A)** and 84.0% in group Z and 90.0% in group P (p=0.609) **(B)**. Group Z, zaltoprofen group; group P, placebo group.

1. An increase in joint circumference was observed in three patients: in two patients in group Z at 48 weeks and in one patient in group P at 12 weeks.

2. The range of motion of joint restriction was not observed.

3. An invasion of ≥5 mm of bone or cartilage erosion or a new lesion of bone or cartilage erosion of ≥5 mm was not observed.

4. The disappearance of joint space was not observed.

5. An increase in the target lesion by ≥20% was determined based on RECIST. An increase in the target lesion judged by the local investigator was observed in two patients in group Z at 24 and 48 weeks and in one patient in group P at 36 weeks (**Figure 2A**). However, those judged by the central committee radiologist were observed in one patient in group Z at 24 weeks and in one patient in group P at 24 weeks (**Figure 2B**). The mean reduction rates of the target lesion were -8.03% in group Z and -2.53 in group P (p=0.271), as judged by the local investigators (**Figure 2C**); these rates were -0.53 in group Z and 3.38 in group P (p=0.292), as determined by the central committee radiologist (**Figure 2D**).

6. PFRs were 84.0% in group Z and 90.0% in group P at 48 weeks (p=0.619) based on any serious event requiring surgical intervention, as judged by local investigators (**Figure 3A**). These rates were 84.0% in group Z and 90.0% in group P (p=0.609), as judged by the central committee radiologist (**Figure 3B**).

#### 3.2.2 Secondary endpoints

**Figure 4:**
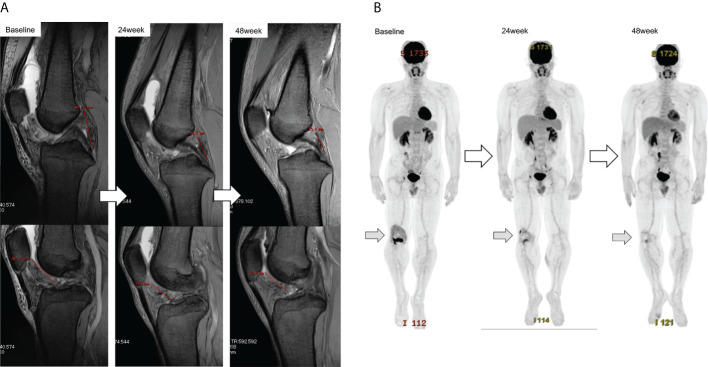
Response of the target tumor size on MRI in patient ZLT-01-08 **(A)**. The longest dimensions judged by the local investigator were 41.5 mm and 40.2 mm at baseline, 30.5 mm and 31.6 mm at 24 weeks, and 25.6 mm and 33.9 mm at 48 weeks. The reduction rates were 15.4% at 24 weeks and 10% at 48 weeks, as judged by the local investigator, and 31.4% at 24 weeks and 30.5% at 48 weeks, as judged by the central committee radiologist. Metabolic response of the target tumor size on FDG-PET in patient ZLT-01-08 **(B)**. Reduction rates were 59.2% at 24 weeks and 70.4% at 48 weeks. MRI, magnetic resonance imaging; FDG-PET, 18F-fluorodeoxyglucose-positron emission tomography.

**Figure 5:**
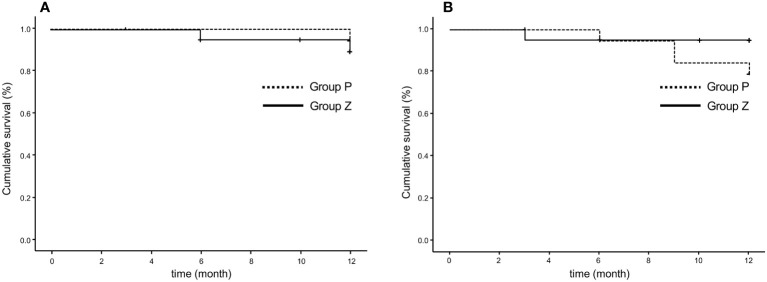
Comprehensive progression-free survival rate based on RECIST (version 1.1), as assessed by the local investigator **(A)** and central committee radiologist **(B)**. The comprehensive progression-free rate at 48 weeks were 89.6% in group Z and 94.7% in group P (p=0.536), as determined by the local investigator **(A)**, and 95.2% in group Z and 78.9% in group P, assessed by the central committee radiologist (p=0.166) **(B)**. Group Z, zaltoprofen group; group P, placebo group; RECIST, Response Evaluation Criteria in Solid Tumors.

1. PFRs determined by the local investigator were 95% and 94.1% in group Z and 100% and 100% in group P at 24 and 48 weeks, respectively, whereas those judged by the central committee radiologist were 95% and 100% in group Z and 94.7% and 83.3% in group P at 24 and 48 weeks, respectively. No significant differences were observed between groups Z and P (**Table 2**; **Figure 4A**). The comprehensive PFRs based on RECIST at 48 weeks determined by the local investigator were 89.6% in group Z and 94.7% in group P (p=0.536), and those assessed by the central committee radiologist were 95.2% in group Z and 78.9% in group P (p=0.166) (**Figure 5**).

**Table 2:** Progression-free rate (24 weeks and 48 weeks).

Judged by the local investigators									
Group	Term (weeks)	n	Response				Progression free rate		p
			CR	PR	SD	PD	n (%)	95%CI	
Z	24	20	0	1	18	1	19 (95%)	75.1- 99.9	1.000 (Z24 vs P24)
	48	17	0	1	15	1	16 (94.1%)	71.3-99.9	0.486 (Z48 vs P48)
P	24	19	0	0	19	0	19 (100%)	82.4-100.0	
	48	18	0	1	17	0	18 (100%)	81.5-100.0	
**Judged by the central committee radiologist**									
Group	Term (weeks)	n	Response				Progression free rate		p
			CR	PR	SD	PD	n (%)	95%CI	
Z	24 48	20	0	1	18	1	19 (95%)	75.1 - 99.9	1.000 (Z24 vs P24)
		17	0	1	16	0	17 (100%)	80.5- 100.0	0.229 (Z48 vs P48)
P	24 48	19	0	0	18	1	18 (94.7%)	74.0 - 99.9	
		18	0	0	15	3	15 (83.3%)	58.6 - 96.4	

CR, complete response; PR, partial response; SD, stable disease; PD, progressive disease.

**Figure 6:**
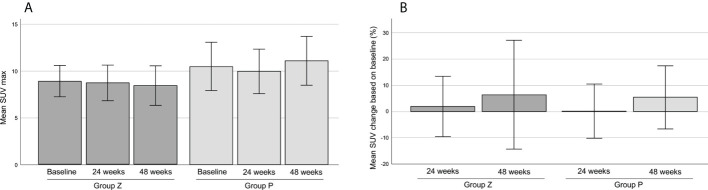
The mean SUVmax on FDG-PET **(A)**. The mean change ratio of SUVmax **(B)**. The mean SUVmax values were 8.88 ± 0.83 (mean ± SE) in group Z and 10.46 ± 1.29 (mean ± SE) in group P at baseline, 8.72 ± 0.95 (mean ± SE) in group Z and 9.94 ± 1.19 (mean ± SE) in group P at 24 weeks, and 8.42 ± 1.06 (mean ± SE) in group Z and 11.07 ± 1.30 (mean ± SE) in group P at 48 weeks **(A)**. The mean change ratios of SUVmax were 1.92% ± 5.75 (mean ± SE) in group Z and 0.08% ± 5.17 (mean ± SE) in group P (p=0.81) at 24 weeks (p=0.818), and 6.35% ± 10.35 (mean ± SE) in group Z and 5.36% ± 6.01 (mean ± SE) in group P (p=0.94) at 48 weeks **(B)**. Group Z, zaltoprofen group; group P, placebo group; FDG-PET, 18F-fluorodeoxyglucose-positron emission tomography; SE, standard error.

2. PET was performed in 21 patients in group Z) and 18 patients in group P. Two patients were excluded from the analysis due to protocol deviations. The mean SUVmax values at baseline were 8.88 (range, 2.4–18.5) in group Z and 10.46 (range, 2.4–25.1) in group P, 8.72 (range, 3.1–17.8) in group Z and 9.94 (range, 2.4–15.9) in group P at 24 weeks; and 8.42 (range, 3.7–20.5) in group Z and 11.07 (range, 2.5–19.2) in group P at 48 weeks (**Figure 6A**). The mean change ratios of SUVmax were 1.92 (range, -61.19–61.82) in group Z and 0.08 (range, -45.45–33.3%) in group P (p=0.81) at 24 weeks (p=0.818) and 6.35% (range, -72.39–86.4%) in group Z and 5.36% (range, -43.18–50.0%) in group P (p=0.94) at 48 weeks (**Figure 6B**). The metabolic response was graded, as shown in **Table 3** (**Figure 4B**).

**Table 3:** Metabolic response of PET.

		Z	%		P	%		
24weeks	PR	3	14.3	90.5%	2 13	12.5 81.3	93.80%	P=0.72
	SD	16	76.2					
	PD	2	9.5		1	6.25		
	total	21			16			
48weeks	PR	3	18.8	81.3%	1 12	5.9 70.6	76.50%	P=0.61
	SD	10	62.5					
	PD	3	18.8		4	23.5		
	total	16			17			

PET, positron emission tomography; CR, complete response; PR, partial response; SD, stable disease; PD, progressive disease.

**Figure 7:**
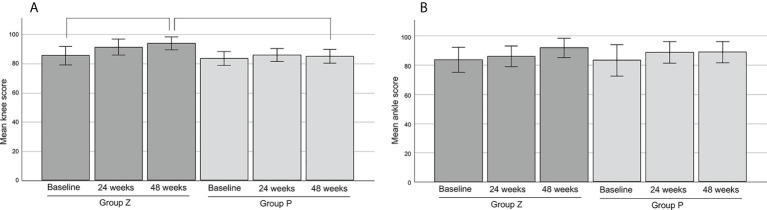
The mean JOA scores of the knee **(A)** and ankle **(B)**. The mean JOA scores of the knee were 85.38 ± 3.12 (mean ± SE) in group Z (n=13) and 83.38 ± 2.33 (mean ± SE) in group P (n=13) at base line. The mean JOA scores of the knee in group Z were 91.15 ± 2.78 (mean ± SE) at 24 weeks and 93.75 ± 2.23 (mean ± SE) at 48 weeks. There was a significant difference between the values at baseline and 48 weeks (p=0.027). The mean JOA scores of the knee in group P were 85.75 ± 2.21 (mean ± SE) at 24 weeks and 85.0 ± 2.38 (mean ± SE) at 48 weeks. There was significant difference between the values at 48 weeks of group P and Z (p=0.014) **(A)**. The mean JOA scores of the ankle were 83.63 ± 4.29 (mean ± SE) in group Z (n=8) and 83.00 ± 5.40 (mean ± SE) in group P (n=7) at baseline. The mean JOA scores of the ankle in group Z were 85.88 ± 3.51 (mean ± SE) at 24 weeks and 91.67 ± 3.35 (mean ± SE) at 48 weeks. The mean JOA scores of the ankle in group P were 88.43 ± 3.70 (mean ± SE) at 24 weeks and 88.71 ± 3.64 (mean ± SE) at 48 weeks. There was no significant difference between each values **(B)**. JOA, Japanese Orthopedic Association; SE, standard error; group Z, zaltoprofen group; group P, placebo group.

**Figure 8:**
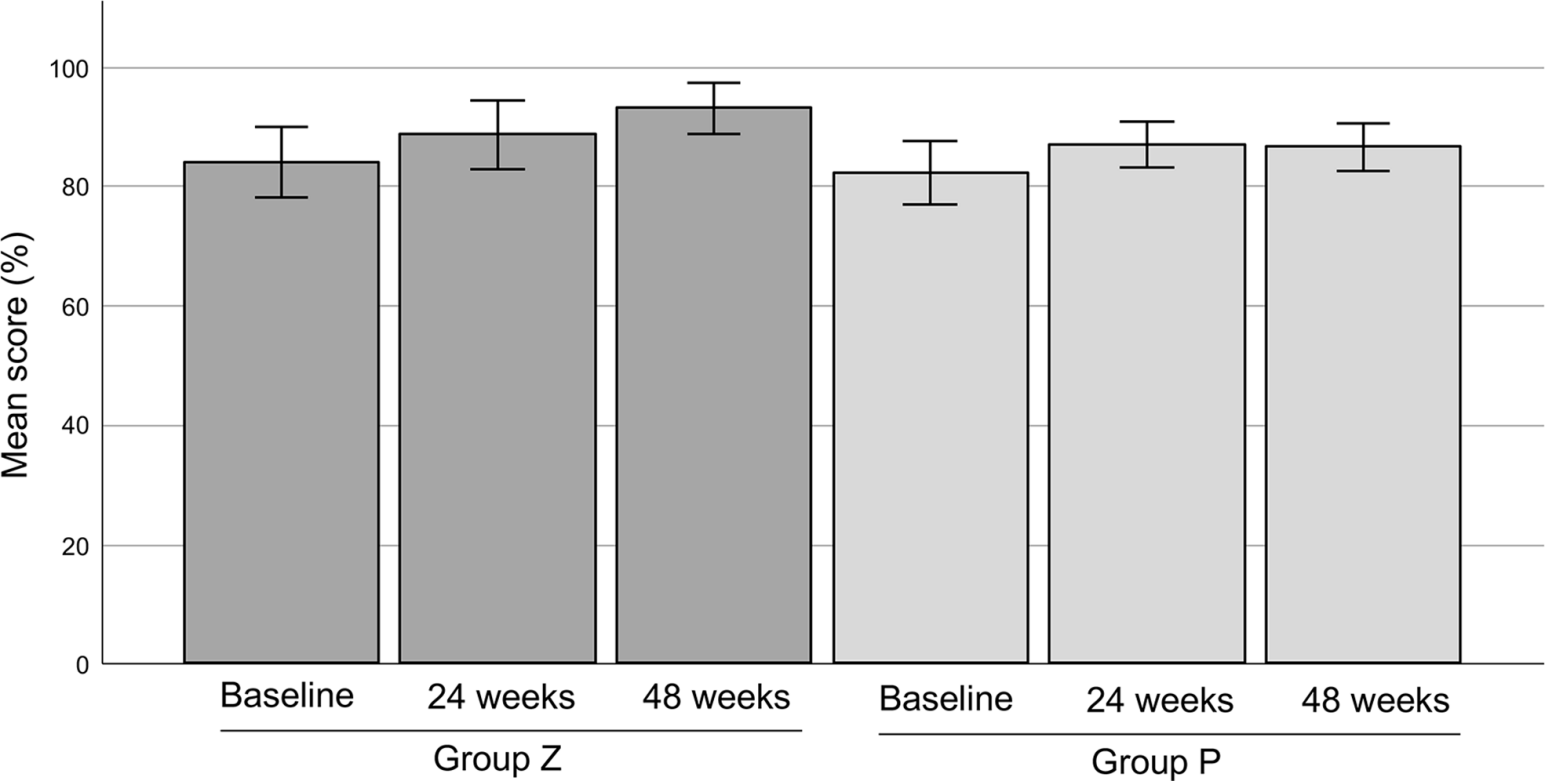
The MSTS scores. The mean MSTS scores were 83.81 ± 2.94% (mean ± SE) in group Z (n=21) and 82.17 ± 2.68% (mean ± SE) in group P (n=20) at baseline. The mean MSTS scores in group Z were 88.57 ± 2.87% (mean ± SE) at 24 weeks and 92.96 ± 2.15% (mean ± SE) at 48 weeks. The mean MSTS scores in group P were 86.84 ± 1.90% (mean ± SE) at 24 weeks and 86.49 ± 2.00% (mean ± SE) at 48 weeks. There was no significant difference between each value. MSTS, Musculoskeletal Tumor Society; SE, standard error; group Z, zaltoprofen group; group P, placebo group.

3. The mean JOA scores of the knee were 85.38 in group Z (n=13) and 83.38 in group P (n=13) at baseline. The mean JOA scores of the knees in group Z were 91.15 at 24 weeks and 93.75 at 48 weeks. There was a significant difference between the values at baseline and 48 weeks (p=0.027). The mean JOA scores of the knees in group P were 85.75 at 24 weeks and 85.0 at 48 weeks. There was significant difference between the values at 48 weeks of group P and Z (p=0.014) (**Figure 7A**).

4. The mean JOA scores of the ankle were 83.63 in group Z (n=8) and 83.00 in group P (n=7) at baseline. The scores were 85.88 (at 24 weeks) and 91.67 (at 48 weeks) in group Z, and 88.43 (at 24 weeks) and 88.71 (at 48 weeks) in group P, respectively. There was no significant difference between each value (**Figure 7B**).

5. The mean MSTS scores were 83.81% in group Z (n=21) and 82.17% in group P (n=20) at baseline. The scores were 88.57% (at 24 weeks) and 92.96% (at 48 weeks) in group Z, and 86.84% (at 24 weeks) and 86.49% (at 48 weeks), respectively. There was no significant difference between each value (**Figure 8**).

6. The proportions of patients with improved clinical benefit in pain, mobility, and function at 24 and 48 weeks of study drug administration compared to that at baseline were nine of 21 cases (42.9%) in group Z (95% confidence interval: 21.8–66.0%) and eight of 20 cases (40.0%) (95% confidence interval: 19.1–63.9%) in group P (p=1.00), and this was only statistically significant when compared between the groups using Fisher’s exact test (**Table 4**).

**Table 4:** Percentage of cases in which clinical benefit regarding pain, range of joint motion, and joint function compared with that of baseline is observed at 24 weeks and 48 weeks.

Group	N	Clinical benefit			p
		Yes	No	95%CI	
Zaltoprofen	21	9 (42.9%)	12 (57.1%)	[21.8 - 66.0]	1
Placebo	20	8 (40.0%)	12 (60.0%)	[19.1 - 63.9]	

7. The incidences of adverse events were 63.6% (14/22) and 73.7% (14/19) in groups Z and P, respectively, and the incidences of adverse reactions were 22.7% (5/22) and 15.8% (3/19), respectively. Adverse events that occurred in two or more cases in either group were infections and viruses (groups Z and P, respectively: 9.1% [2/22 cases], 5.3% [1/19 cases]), stomatitis (9.1% [2/22 cases], 0% [0/19 cases]), viral upper respiratory tract infection (13.6% [3/22 cases], 15.8% [3/19 cases]), and muscle pain (9.1% [2/22 cases], 0% [0/19 cases]). There was no significant difference in the incidence of adverse events between the groups. No adverse events leading to death, serious adverse events, or discontinuation or withdrawal of the study drug were observed. No grade 5 or grade 4 adverse events were observed. The only grade 3 adverse event was hypertension in group Z, and a causal relationship with the study drug was not ruled out (**Supplementary Table 3**).

## 4 Discussion

To our knowledge, this is the first study to evaluate the efficacy of zaltoprofen in patients with TGCT. No significant differences in PFR were observed between the groups at 48 weeks. Group P also tended to maintain SD during this period. Zaltoprofen moderately improved the physical function of the affected limb, especially knee function. This appears to be a novel approach for managing TGCT. Zaltoprofen was also safely tolerated by the patients.

Based on a previous pilot study (24), we estimated that 80% of patients treated with zaltoprofen would maintain SD (25). It is difficult to speculate on the progression rate of untreated TGCT. Therefore, we defined the original criteria to reflect tumor progression. Finally, we expected the PFRs to be 80% and 30% in groups Z and P, respectively. However, both groups presented with an SD course for 48 weeks. There were only three events (one patient with increase in lesion size, one with increase in joint circumference, and one with both) in group Z and two events (one patient with increase in lesion size and one with increase in joint circumference) in group P. There was no significant difference in PFR between the groups. According to the original criteria, only the size of the target lesion was evaluated; however, the mean size at the baseline showed a discrepancy between the investigator and the central reviewer. Regarding the change in tumor size in group P, the mean change at 48 weeks decreased, as judged by the investigator, whereas it increased, as judged by the central reviewer. This reflects the difficulty in evaluating the exact tumor size and changes in TGCT. Some studies on TGCT evaluated the tumor volume score (TVS) and mentioned the possibility of precise tumor volume evaluation compared with RECIST (8, 30). Overall, PFR in group Z was similar to that expected, whereas that in group P was extremely high. The natural progression rate, as reported in another study, showed that the mean best shrinkage was 3.4% (**Figure 2**), with three cases of PD (15%) based on RECIST (**Table 1**), as judged by the central reviewer. In another study on pexidartinib (the ENLIVEN study), the progression rates in the placebo group were 2% (one of 59 cases) based on RECIST and 3% (2 of 59 patients) based on TVS at 25 weeks (8). Recently, a prospective cohort study of TGCT was registered, which is expected to reveal the natural course of TGCT (13).

We evaluated the following secondary endpoints: PFR based on RECIST, PET findings, limb function, and the clinical benefit. PFR was evaluated based on RECIST, which included non-target and new lesions. The central committee radiologist judged three cases of PD in group P and none in group Z at 48 weeks. PFR based on RECIST at 48 weeks was 95.2% in group Z and 78.9% in group P (p=0.166). Although there was no significant difference between the groups, the survival curve in group P declined slightly in a time-dependent manner. Therefore, a more long-term evaluation appears to be the next step with other criteria, such as total tumor volume.

The SUVmax of TGCT on PET has been reported as high (31). The mean SUVmax in our study was high at 9.6 (range, 2.4–25.1) at baseline, and there was no significant difference between the groups. The mean change in SUVmax gradually increased, and the metabolic response showed that the incidence of PD gradually increased in both groups. Other parameters, such as total lesion glycolysis and peak standardized uptake normalized to lean body mass, have been reported as useful predictors of clinical outcomes in certain cancers (32, 33). Further analyses including various parameters are necessary. TGCT can develop in uncommon sites and is incidentally detected *via* PET scan (34, 35). TGCT have a high SUVmax, even when the tumor is benign, which can be used to prevent misdiagnosis of malignancy.

We evaluated the physical limb function using the JOA and MSTS scores. The JOA score has also been reported to correlate with patient-reported outcomes (Japanese Knee Osteoarthritis Measure and 36-Item Short Form Health Survey [SF-36]) (36). This study revealed a significantly higher score at 48 weeks than at baseline in group Z, while no significant difference between the JOA Foot Rating Scale values and MSTS score at baseline and after 48 weeks was observed. However, the score gradually increased in a time-dependent manner. Palmerini et al. reported a median MSTS score of 90% (range, 20–100%) after surgical tumor resection (37). Griffin et al. reported a mean score of 91.7% (range, 63–100%) after radiotherapy and surgery (38). Our results are comparable with those of such invasive treatments. Kask reported that the MSTS score correlated with the Toronto Extremity Salvage Score of patient-reported outcomes in lower extremity soft-tissue sarcomas (39).

Patients with TGCT usually have pain, joint swelling, stiffness, reduced range of motion, and joint instability, which diminish the joint function (40), therefore, the amelioration of physical function is beneficial for such patients with TGCT. Sande et al. also reported that physical function improved after pexidartinib treatment (41). Several reports have also described favorable limb function after surgery (42, 43). However, surgery is not a definitive treatment for every patient since it is associated with high risk of local recurrent disease due to diffuse lesions and a relatively high risk of postoperative complications (44).

Our results also suggest that the zaltoprofen is safe. Several drugs targeting the CSF-1 and CSF-1R axis have shown some severe side effects (8, 45–47). For instance, emactuzumab was tested in a phase 1 study that enrolled 29 patients. The authors reported three cases of serious adverse events (10.3%) (one case of periorbital edema, one case of subacute cutaneous lupus erythematosus, and one case of subcutaneous tissue inflammation) (45). A single-arm phase 2 study of nilotinib, a small-molecule inhibitor, was conducted in 56 patients, and serious adverse reactions were reported, including pruritus, diarrhea, anorexia, dizziness, toxic skin rash, and liver enzyme abnormalities in nine patients (16.7%) each (46). In a phase 1 study of pexidartinib, fatigue, diarrhea, anemia, and neutropenia were observed in one case each and hyponatremia and aspartate aminotransferase (AST)/alanine transaminase (ALT) elevation were observed in two cases each (47). In a phase 3 study of pexidartinib, 61 patients in the placebo group and 59 in the pexidartinib group were enrolled. Serious adverse events were observed in 32 patients in the pexidartinib group (52.5%) (one case each of erythema, vomiting, lactate dehydrogenase elevation, dizziness, periorbital edema, anemia, and neutropenia; two cases each of arthralgia, hyponatremia, and AST/ALT elevation; three cases of hypertension; four cases of alkaline phosphatase elevation; and six cases of AST and ALT elevation) (8).

This study has some limitations. The number of patients was small, with a heterogeneous background. Patient status (primary or recurrent) and the number of previous surgeries were not considered in the randomization. Patient-reported outcomes were not evaluated. The SF-36, Western Ontario and McMaster Universities Arthritis Index, and patient-reported outcome measures (42) might be suitable for evaluating the improvement in patient quality of life and physical function.

In conclusion, our study reported no significant differences in PFR between the groups. Physical function significantly improved following zaltoprofen treatment. The safety profile of zaltoprofen was acceptable. Both groups presented with an SD course for 48 weeks; therefore, the long-term clinical course of TGCT should be clarified. This less invasive and safer treatment, which improved physical function in patients with SD with zaltoprofen, is a potential novel approach against TGCT. Further analysis of long-term administration of zaltoprofen should be considered in future studies.

## Data availability statement

The data that support the findings of this study are available from HT, tsuchi@med.kanazawa-u.ac.jp, upon reasonable request

## Ethics statement

This study was reviewed and approved by the Ethics Committees of Kanazawa University Hospital (9013), Asahikawa Medical University Hospital (29024), National Cancer Center Hospital (T4456), University of Fukui Hospital (2017008), Nagoya City University Hospital (31-17-0003), Nagoya University Hospital (29005), Mie University Hospital (F2913012), Kyoto Prefectural University Hospital (2017– 011), Osaka City University Hospital (101952), Okayama University Hospital (290801) and Kyushu University Hospital (2017301). The patients/participants provided their written informed consent to participate in this study.

## Author contributions

AT and HT conceived of the study and drafted the manuscript. AT, AN, TM, and HT participated in the study design, and KY was responsible for the design of the statistics. HT was the principal investigator of this study. AT, ME, AK, YN, RT, AM, HA, TN, ST, TO, and MH contributed with the local investigator. MO reviewed all magnetic resonance imaging and computed tomography scans as part of the central committee. DK reviewed all ^18^F-fluorodeoxyglucose- positron emission tomography scans as part of the central committee. AT performed the statistical analyses and KY reviewed the analyses. AT, AN, and TM wrote the manuscript. NY, KH, KI, and SM provided critical comments on the study protocol design and helped draft the manuscript. All authors contributed to the article and approved the submitted version.

## Funding

This research was partially supported by the Project Promoting Clinical Trials for Development of New Drugs and Medical Devices (Japan Medical Association) (JMAIIA00284) and the Japan Agency for Medical Research and Development (21lk0201120h0002). This funding body did not support the design of the study; collection, analysis, and interpretation of data; or writing of the manuscript.

## Acknowledgments

We gratefully acknowledge the help of the study coordinators and physicians at each institution and the central committee: Hiroaki Shibata, Shintaro Iwata, Yuya Izubuchi, Tsuyoshi Miyazaki, Yasuro Kokubo, Takehiro Ota, Toshiyuki Kunisada, Joe Hasei, Toshiharu Shirai, Akihiko Iida, Hiroaki Kimura, Naoto Oebisu, Kunihiro Asanuma, Yoshihiro Matsumoto, Masako Yamazaki, Mitsuyo Kusajima, Kazuto Kosaka, Takashi Higuchi, Kensaku Abe, Yuta Taniguchi, Hirotaka Yonezawa, Sei Morinaga, Yohei Asano, Takafumi Ueda, Junichi Taki, and Satoshi Teramukai. Nippon Chemiphar provided the study medication, zaltoprofen, and the placebo.

## Conflict of interest

HT received research funding from Nippon Chemiphar.

The remaining authors declare that the research was conducted in the absence of any commercial or financial relationships that could be construed as a potential conflict of interest.

## Publisher’s note

All claims expressed in this article are solely those of the authors and do not necessarily represent those of their affiliated organizations, or those of the publisher, the editors and the reviewers. Any product that may be evaluated in this article, or claim that may be made by its manufacturer, is not guaranteed or endorsed by the publisher.
